# Light‐Intensity Switching of Graphene/WSe_2_ Synaptic Devices

**DOI:** 10.1002/advs.202309876

**Published:** 2024-04-22

**Authors:** Hongyu Tang, Tarique Anwar, Min Seok Jang, Giulia Tagliabue

**Affiliations:** ^1^ Laboratory of Nanoscience for Energy Technologies (LNET) École Polytechnique Fédérale de Lausanne Station 9 Lausanne CH‐1015 Switzerland; ^2^ School of Electrical Engineering Korea Advanced Institute of Science and Technology Daejeon 34141 Republic of Korea; ^3^ Present address: Academy of Engineering & Technology, Fudan University Handan Road 220 Shanghai 200433 China

**Keywords:** graphene/WSe_2_ van der Waals heterojunction, optoelectronic synapses, photogating, phototransistors

## Abstract

2D van der Waals heterojunctions (vdWH) have emerged as an attractive platform for the realization of optoelectronic synaptic devices, which are critical for energy‐efficient computing systems. Photogating induced by charge traps at the interfaces indeed results in ultrahigh responsivity and tunable photoconductance. Yet, optical potentiation and depression remain mostly modulated by gate bias, requiring relatively high energy inputs. Thus, advanced all‐optical synapse switching strategies are still needed. In this work, a reversible switching between positive photoconductivity (PPC) and negative photoconductivity (NPC) is achieved in graphene/WSe_2_ vdWH solely through light‐intensity modulation. Consequently, the graphene/WSe_2_ synaptic device shows tunable optical potentiation and depression behavior with an ultralow power consumption of 127 aJ. The study further unravels the complex interplay of gate bias and incident light power in determining the sign and magnitude of the photocurrent, showing the critical role of charge trapping and photogating at interfaces. Interestingly, it is found that switching between PPC to NPC can be also obtained at 0 mV drain‐source voltage. Overall, the reversible potentiation/depression effect based on light intensity modulation and its combination with additional gate bias tunability is very appealing for the development of energy‐efficient optical communications and neuromorphic computing.

## Graphical abstract

A graphene/WSe2 device exhibits switching between positive/negative photoconductivity solely upon changing the illuminating light intensity. A proof‐of‐concept synaptic device with extremely low power consumption is demonstrated.

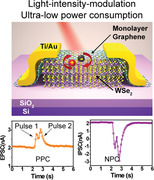

## 1 Introduction

Graphene (Gr) and transition metal dichalcogenides have emerged as excellent materials for optoelectronic devices thanks to high carrier mobility^1^ and strong light absorption across the visible range,^[^ ^1^ ^]^ respectively. Because of the absence of dangling bonds on their surfaces, graphene and transition metal dichalcogenides can also be freely stacked to form van der Waals heterojunctions (vdWH), which can effectively harness photons and exhibit ultrahigh responsivity.^[^ ^2^ ^]^ However, photoexcited carriers can get captured by local potential fluctuations on the trap sites of vdWHs, inhibiting fast recombination and limiting the device response time.^[^ ^3^ ^]^ While extremely detrimental for photodetection applications, this mechanism opens new opportunities toward vdWHs applications in the field of neuromorphic devices, such as synaptic devices and brain‐like computing chips.^[^ ^4^ ^]^ In fact, trapped charges result in a photogating effect, i.e., a shift in the Fermi level of graphene.^[^ ^5^ ^]^ This can be leveraged to optically modulate the sign and magnitude of the photoconductivity, in principle eliminating the need for an external gate bias and minimizing the overall energy consumption.

Current photosynapses based on vdWHs are mostly three‐terminal devices, where the photoresponse is controlled through the gate bias.^[^ ^6^ ^]^ Pradhan et al.^[^ ^7^ ^]^ used graphene‐perovskite quantum dot hybrids as the basis for optoelectronic synapses in the field of facial recognition. In the reported device photogenerated electrons accumulate in the perovskite quantum dots. This results in photogating and produces a hole current in the graphene channel. In this case, the dominant carrier type in the channel is modulated through the gate voltage. Yu et al.^[^ ^8^ ^]^ proposed a bioinspired mechano‐photonic artificial synapse which is composed of a Gr/MoS_2_ heterostructures‐based phototransistor and an integrated triboelectric nanogenerator, the latter modulating the optoelectronic synaptic behavior. Kim et al.^[^ ^9^ ^]^ fabricated a MoS_2_/hBN/WSe_2_/Gr gate‐tunable optoelectronic synapse device based on the tunneling of electrons or holes between the WSe_2_ channel and the floating gate. The devices performed learning and recognition tasks by using the artificial neural network based on a single‐layer perceptron model and the recognition rate. Sun et al.^[^ ^10^ ^]^ constructed an asymmetric WSe_2_/Gr heterostructure to achieve optical modulation of hysteretic behavior. The results showed that inhibitory and excitatory synapses can be switched by changing the polarity of the electrical spikes through gate bias.

Overall, to date, the photogating effect was used to modulate the carrier concentration in the vdWH by changing the intensity of the light. Most of the cases show that the carrier concentration increases monotonously with illumination intensity, while electrical stimuli can modulate the charge transfer and determine the sign of the photocurrent. Therefore, optical stimulation is used for synaptic potentiation while electrical stimulation is required for synaptic depression.^[^ ^11^ ^]^ However, electrical stimuli result in additional energy consumption and limit the integration of complex 2D materials‐based devices, which is a significant disadvantage of optoelectronic synapses. Therefore, it is necessary to integrate optical potentiation and depression within one device, using a single optical stimulus.

In this paper, we report an optoelectronic synaptic device based on Gr/WSe_2_ vdWH that exhibits tunable optical potentiation and depression performance simply by adjusting the incident light power. This is achieved thanks to charge trapping at different interfaces present in the device. The photogating mechanism is systematically analyzed considering the shift of the Dirac point, the photoconductivity flipping point, and the role of impurities density. It is found that the gate bias and laser power can modulate the photogating level as well as the sign and magnitude of the photocurrent. Besides, this optoelectrical synapse successfully carried out typical synaptic functions, including excitatory postsynaptic current (EPSC), inhibitory postsynaptic current (IPSC), and paired‐pulse facilitation (PPF). In addition, the synaptic plasticity was successfully modulated by varying the parameters of light spikes. It is worth noting that it has an extremely low electrical power consumption of ≈127 aJ. This work provides a strategy to fabricate fully optical‐modulated neuromorphic devices.

## 2 Results and Discussion

**Figure 1:**
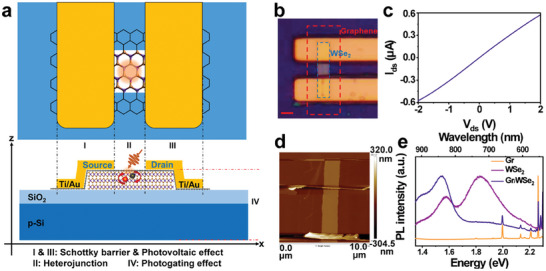
a) Schematic of the Gr/WSe_2_ heterojunction‐based device. b) Optical image of Gr/WSe_2_ heterojunction‐based device. The scale bar indicates 5 µm. c) Current–voltage characteristics of the device under dark. d) The AFM image of the heterojunction, and e) PL spectra of the pristine graphene (orange), the pristine WSe_2_ (purple), and Gr/WSe_2_ heterojunction (royal) at room temperature.

The Gr/WSe_2_ three‐terminal device is constructed by first dry‐transferring a WSe_2_ multi‐layer flake onto a SiO_2_/Si substrate, where Si can be used to apply a gate bias (V_gs_). Subsequently, the WSe_2_ flake is etched into a 15 µm × 5 µm rectangle. Next, monolayer graphene (Graphenea, Inc.) is wet‐transferred over the WSe_2_ patch and etched into a ribbon with length and width of 20 and 10 µm, respectively (see **Figure** **1**a,b and Experimental Section). Finally, source (s) and drain (d) Ti/Au contacts are evaporated to contact the graphene ribbon. With this device geometry, graphene short‐circuits WSe_2,_ and it is possible to minimize the photoresponse which is otherwise induced by the distinct Schottky barriers created at the Gr/Au and WSe_2_/Au contacts. The current–voltage characteristics of the device in the dark (Figure 1c), measured as the source‐drain current, I_ds_, as a function of the source‐drain voltage, V_ds_, show a weak rectification behavior in the high voltage section (1 to 2 V), caused by the Schottky contact between graphene and Ti/Au. An atomic force microscopy (AFM) image of the Gr/WSe_2_ device is shown in Figure 1d. The thickness of the WSe_2_ flake is 5 nm. The surface of the device is flat and uniform. Photoluminescence spectroscopy in Figure 1e reveals that, from 1.2 to 2 eV, light emission is quenched in the heterojunction compared to pristine WSe_2_. This indicates that charges in the interlayer were coupled between graphene and WSe_2_ in the wavelength range of 750–620 nm.^[^ ^12^ ^]^

**Figure 2:**
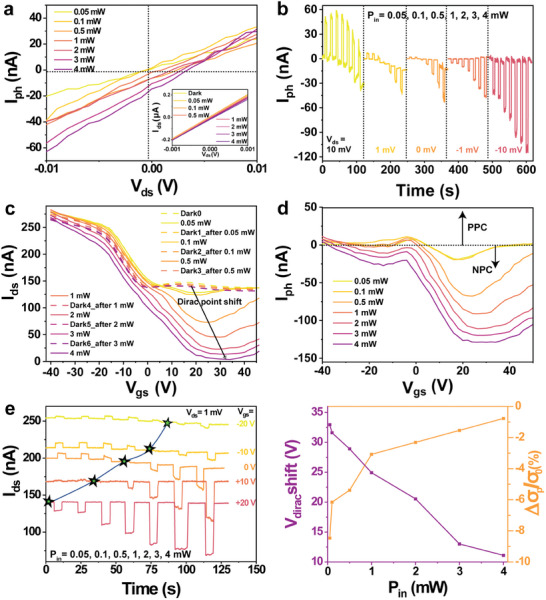
a) The photocurrent (I_ph_) of the Gr/WSe_2_ heterojunction‐based device under illumination (635 nm, 0.05‐4 mW) at V_gs_ = 0. The inset enlarged image shows the output curve from −0.1 to 0.1 mV. b) Dynamic photocurrent current change at V_gs_ = 0 V and different P_in_ for a series of V_ds_. c) Transfer characteristics of the Gr/WSe_2_ device at different illumination powers (solid lines) and in the dark after each illumination condition (dash lines). It shows the shift of the Dirac point for a constant V_ds_ = 1 mV. d) The photocurrent of the Gr/WSe_2_ device at different illumination power. e) Dynamic current change at different P_in_ after applying different V_gs_ for a constant V_ds_ = 1 mV. f) Shift of Dirac point and the fractional change in dark conductivity (at zero gate bias) after different P_in_.

To avoid any photoinduced carriers generated in the Gr/Ti/Au junction region, we used a laser source with a 4.4 µm illumination spot diameter located at the center of the heterojunction. Overall, in this structure graphene works as the carrier transport channel and WSe_2_ contributes the electron–hole pairs under illumination. Under illumination, several distinct effects are expected to play a role in determining the overall device photoresponse. First of all, any unavoidable asymmetry between the source/drain Gr/Ti/Au contacts^[^ ^13^ ^]^ creates a built‐in potential and therefore a source/drain photovoltage under illumination (see Region I, II, and IV in Figure 1a). Furthermore, defects and impurities in vdWHs can act as trap centers, resulting in photogating effects^[^ ^14^ ^]^ (see Region III and IV in Figure 1a). Thus, two photogating contributions are expected in this system, originating from the Gr/WSe_2_ vdWHs and WSe_2_‐SiO_2_‐Si^[^ ^15^ ^]^ and Gr‐SiO_2_‐Si^[^ ^16^ ^]^ interfaces, respectively. Importantly, the relative band position of each interface in different operating conditions (gate bias and laser power) will determine the net photogating effect. Overall, it is the interplay of these different effects that determines the sign of the photocurrent and whether illumination results in potentiation or depression of the device (shown in **Figure** **2**). Thus, we first characterize the device photoresponse in a broad range of operating conditions and subsequently demonstrate its operation as a synaptic device controlled solely by the laser intensity.

### 2.1 Optoelectronic Response of Graphene/WSe_2_ Devices

The current‐voltage curves of the Gr/WSe_2_ device under illumination and in the dark are shown in Figure S1 (Supporting Information). Here, the photocurrent (I_ph_) in the Gr/WSe_2_ device is defined as the difference between the source‐drain currents under illumination and in the dark, i.e., I_ph_ = I_Light_ − I_Dark_. As shown in Figure 2a, at V_gs_ = 0 V and under 635 nm illumination, I_ph_ varies with increasing incident laser power (P_in_). Interestingly, in Figure 2a, when V_ds_ is zero, I_ph_ is negative. This can be associated with the unavoidable asymmetries at the two metal/graphene contacts resulting in a small built‐in electrical potential difference (≈0.2 mV, see Figure S2, Supporting Information).^[^ ^17^ ^]^ Furthermore, under illumination, a non‐zero photovoltage is generated at each metal/graphene contact,^[^ ^18^ ^]^ which is estimated in the range of 0.4–0.6 mV (Figure S2; Section S1, Supporting Information). Overall, it is possible to define an effective drain‐source voltage ($V_{\mathrm{ds}}^{\mathrm{eff}}$) under illumination as:

(1) $$V_{\mathrm{ds}}^{\mathrm{eff}}=V_{\mathrm{ds}}+V_{\mathrm{PV}}$$

The sign of $V_{\mathrm{ds}}^{\mathrm{eff}}$ can be positive or negative depending on the interplay of the different terms. Since the graphene channel is p‐doped, the photocurrent at V_gs_ = 0 V can be given by

(2) $$I_{\mathrm{pc}}=\frac{e\mu_{p}p^{*}AV_{\mathrm{ds}}^{\mathrm{eff}}}{L}$$

where µ_p_, *p* ^*^, *A*, and *L* are the hole mobility, photo‐generated carriers, area, and length of the graphene channel, respectively. Therefore, its sign depends on $V_{\mathrm{ds}}^{\mathrm{eff}}$ and the type of photogenerated carriers. Next, we applied a series of laser pulses with a duration of 5 s and increasing P_in_, from 0.05 to 4 mW to the device. The pulse interval was 10 s. As shown in Figure 2b, for V_gs_ = 0 V, changing V_ds_ from 10 to ‐10 mV. Interestingly, when V_ds_ is small and positive (red curve), increasing P_in_ results in a change from positive photoconductivity (PPC) to negative photoconductivity (NPC). From further testing, we find that when V_ds_ ≤ −0.029 mV, only NPC is obtained (Figure S3, Supporting Information), which means $V_{\mathrm{ds}}^{\mathrm{eff}}\;$< 0 V. Notably, when a very small V_ds_ is applied, the response time of the device slows down and a synaptic behavior occurs, which we will discuss in Section 2.2. Furthermore, as shown in Figure S4 (Supporting Information), the second pulse does not affect the first one when there is a time gap, longer than 3 s between consecutive illumination.

**Figure 3:**
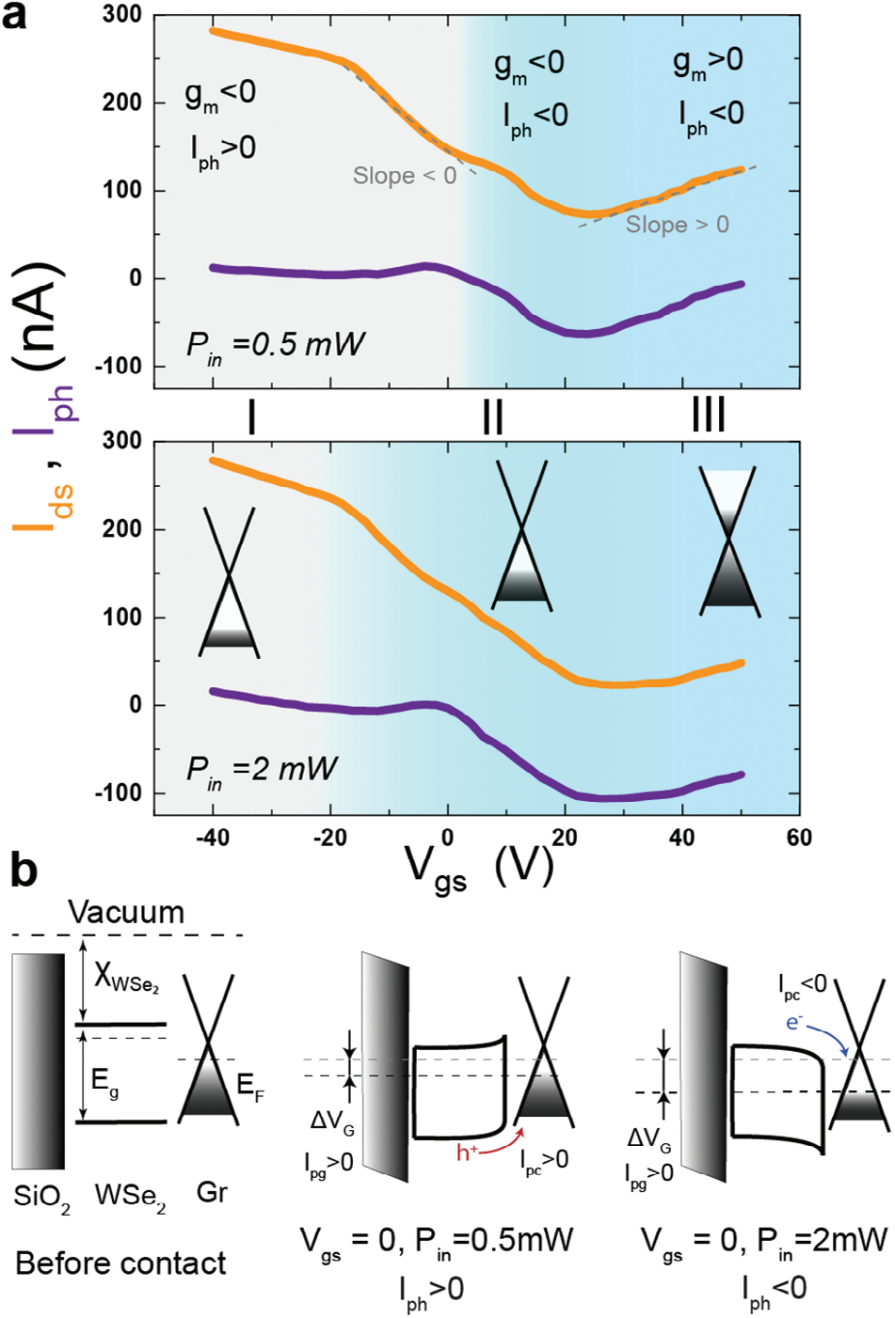
Mechanism for flipping in the sign of the photocurrent in Gr/WSe_2_ heterojunction by changing V_gs_ and P_in_. a) Drain source current and photocurrent (I_ph_)with varying V_gs_ and different applied P_in_, 0.5 mW (top panel) and 2 mW (bottom panel). Three different regions have been identified depending on the sign of transconductance (g_m_) and net I_ph_. PPC is observed in region I, while regions II and III show NPC. The photogenerated carriers injected from WSe_2_ give rise to the photoconductive component of the resultant photocurrent. At high power this becomes more negative and grows in magnitude, thus broadening the region II. b) Band diagram for three different conditions: (left panel) before contact, X_WSe2_ is the WSe_2_ electron affinity, *E*_g_ is the WSe_2_ bandgap, *E*_F_ is the fermi level of graphene; (middle panel) band alignment for *V*_GS_ = 0 and *P*_in_ = 0.5 mW (region I): I_pg_ > 0 while the upward band‐bending at the WSe_2_/Gr interface results in I_pc_ > 0 (hole injection), overall leading to I_ph_ > 0 (PPC); (right panel) band alignment for V_GS_ = 0 and P_in_ = 2 mW (region II): I_pg_ > 0 while the downward band‐bending at the WSe_2_/Gr interface, caused by the larger photovoltage, results in I_pc_ < 0 (electron injection), overall leading to I_ph_ < 0 (NPC). I_pg_ and I_pc_ are the photogating and photoconductive components of the photocurrent.

In addition to the effects of photovoltage and built‐in electrical potential difference within the source/drain channel, the photogating effect originating from the Gr/WSe_2_ vdWHs and Gr‐WSe_2_‐SiO_2_‐Si interfaces affects the photoresponse in this system. In particular, it is known that in heterojunctions with low dimensional materials, defects and impurities in the structure can work as trap centers.^[^ ^14^ ^]^ If one type of photogenerated carrier is trapped for a time *τ*, which is greater than *τ =* L^2^/µ*V*_ds_, the average time for the carriers to move through the graphene channel,^[^ ^14^ ^]^ carrier accumulation will occur at the interface providing an additional gate voltage, ΔV_G_. To assess the role of photogating, we applied different gate biases (V_gs_) to the device and measured the transfer curves of the device for different P_in_ (Figure 2c). As the P_in_ increases, the Dirac point shifts to higher V_gs_, suggesting a negative gating (ΔV_G_ < 0) caused by trapped electrons in trap centers. Here, the photocurrent generated by the photogating effect can be expressed as^[^ ^14^ ^]^

(3) $$I_{pg}=g_{m}\Delta V_{G}$$

where g_m_ is the transconductance. ΔV_G_ is the horizontal displacement of the transfer characteristic curve. As shown in Figure 2d, the device shows gate‐tunable positive and negative photoresponse. When V_gs_ < V_Dirac_dark_, the majority carrier in dark is the holes (g_m_ < 0), thus according to Equation (3), it shows PPC (I_ph_ > 0). While at V_gs_ > V_Dirac_dark_, the majority carrier in dark is electron (g_m_ > 0), NPC occurs (I_ph_ < 0). However, there exists a regime where both g_m_ and I_ph_ are negative due to photoconductive components as shown in **Figure** **3**. Furthermore, the peak photocurrent is associated with the V_Dirac_, because the device reaches a high electrostatic doping with either electrons or holes.^[^ ^19^ ^]^

The dynamic current responses of the device for different gate biases under various laser power illuminations are shown in Figure 2e. For V_gs_ < 0 V, the device initially exhibits PPC. As P_in_ increases, the photoconductivity flips to negative. It is important to note that the photodoping effect is not sufficient to explain the switchable photocurrent of this device. As shown in Figure S5 (Supporting Information), it is found that the I_ds_‐V_gs_ shift under illumination is induced by both photogating and photoconductive effects. Indeed, I_ds_‐V_gs_ not only shifts to the right but also moves down. The ΔV_G_ shifts to higher V_gs_ corresponds to graphene becoming deeply p‐doped, with a down‐shift of its E_F_. Normally, this entails a photocurrent increase as many holes move into graphene. However, in our case, the photocurrent decreases with increasing P_in_, which is then most likely related to the n‐doping effect from impurities.

We further note that the dark current/gate‐bias curve after each illumination cycle is shifted left and downwards compared to the previous dark transfer curve (see dash lines in Figure 2c and blue line in Figure S5, Supporting Information). It reveals that the trapped carriers are not immediately released when the light is turned off. This can be related to changes in the impurity density (*n_imp_*) within the substrate.^[^ ^20^ ^]^ This effect is very pronounced in our device because of the very low initial doping of graphene and thus comparable to changes in *n_imp_*, as shown by the close to zero Dirac point (dashed lines in Figure 2c). Indeed, we modeled the effect of an increase in *n_imp_* on the transfer curve in the dark (Figure S6 and Section S4, Supporting Information), retrieving qualitatively the experimental trend. As the impurity density directly affects the carrier mobility in graphene,^[^ ^21^ ^]^ (µ∝1/*n_imp_*) and in turn the dark conductivity (σ_d_), we can use the experimental I_ds_–V_ds_ dark curves (Figure S1, Supporting Information), to estimate the fractional change in σ_d_ after each illumination as $\frac{\Delta \sigma_{d}}{\sigma_{0}}=\frac{I_{\mathrm{Dark}(i)}-I_{\mathrm{Dark}0}}{I_{\mathrm{Dark}0}}\;$. From Figure 2f (orange curve), we find that the conductivity in the dark decreases with increasing P_in_. This also affects the photoconductive component of the resulting photocurrent as, *I*_pc_∝(Δσ_*d*_ + Δσ), where, Δσ is the photoinduced change in conductivity. For V_ds_ = 1mv, the sign of $V_{\mathrm{ds}}^{\mathrm{eff}}$ does not change so, Equation (2) can be equivalently written as $\;\frac{\;I_{\mathrm{pc}}}{I_{Dark0}}\approx \;\frac{\Delta \sigma_{d}}{\sigma_{0}}\pm \frac{\Delta p}{p_{0}}$, where Δ*p* is the photogenerated carriers injected into the graphene channel. Thus for the case, where Δσ_*d*_ ≈ 0, a positive (negative) photocurrent is observed if the carriers injected into the channel are the same (opposite) to the majority carrier in the channel.^[^ ^14^ ^]^

We note that at high power this would grow in magnitude due to an increase in photogenerated carriers in WSe_2_ that can be injected into graphene. We also observed that the effect exhibits a long recovery time, which can be important for neuromorphic applications of the device discussed in Section 2.2. In addition, we interestingly observe that when the P_in_ reaches 1 mW, the Dirac point shift slows down, implying that the photogating effects become saturated (Figure 2f, purple curve). This is a typical behavior related to the gradual filling of trap states with increasing laser power.^[^ ^22^ ^]^

To better understand the tunable photoconductance of the device, we have identified three different regimes (I: I_ph_> 0, g_m_ < 0; II: I_ph_ < 0, g_m_ <0; III: I_ph_ < 0, g_m_ > 0) in the transfer curve under different illumination conditions as shown in **Figure** **3**a. Based on the shift of the transfer curve under illumination, as discussed above, we know that trapped charges induce a negative photovoltage (ΔV_G_ < 0), i.e., increased p‐doping of graphene. Hence the sign of the photogating component of the current will depend on the transconductance of the transfer curve, specifically I_pg_ > 0 if g_m_ < 0 and vice versa. Looking at the orange curves in Figure 3a, we can thus conclude that I_pg_ > 0 for regimes I and II. On the other hand, the sign of the photoconductive current, I_pc_, will be positive (negative) if majority (minority) carriers are injected. In regions I and II of Figure 3, graphene is p‐doped. Therefore, I_pc_ > 0 if holes are injected and I_pc_ < 0 if electrons are injected. To determine which condition is occurring, we observe that in region II of Figure 3, the total sign of the photocurrent is negative. As in this regime, the photogating component is positive; the photoconductive term must be negative, meaning that electrons are injected into the p‐doped graphene. Finally, from Figure 3a we note that region II broadens when going from weaker (P_in_ = 0.5 mW) to stronger (P_in_ = 2 mW) illumination intensities. Importantly, for a certain range of V_gs_, including V_gs_ = 0, the sign of I_ph_ flips as the intensity of illumination increases. Overall, considering V_gs_ = 0 as our reference case, we can draw the band alignment depicted in Figure 3b. Under weak illumination (middle panel), the upward band‐bending at the Gr/WeS_2_ interface allows injection of holes in the p‐doped graphene, giving a positive I_pc_ that together with a positive I_pg_ results in PPC, i.e., I_ph_ > 0. However, as the intensity of illumination is increased, the larger photovoltage causes a flipping of the band‐bending. Hence, photogenerated electrons in WSe_2_ preferentially transfer to graphene, giving a negative I_pc_ that overcomes the positive I_pg_ to produce NPC, i.e., I_ph_ < 0.

### 2.2 Neuromorphic Optoelectronic Synapse

**Figure 4:**
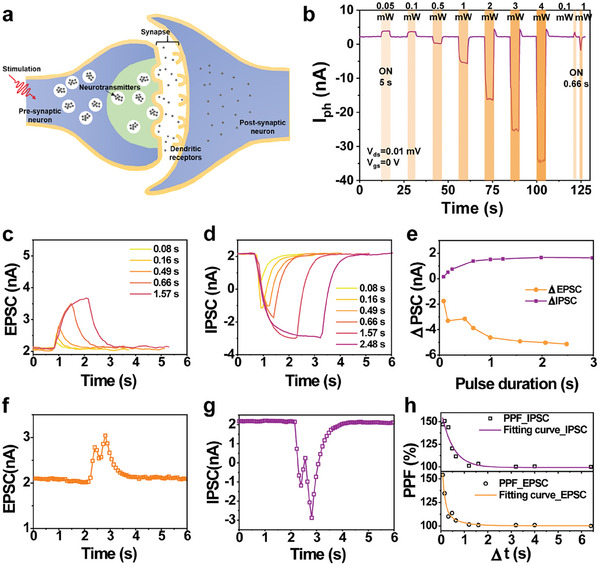
a) Schematic Diagram of signal transmission in biological synapses b) Dynamic change of I_ds_ after illumination with different laser power at different laser pulse duration, V_gs_ = 0 V, V_ds_ = 0.01 mV. c) EPSC induced by the light spike of fixed light intensity (635 nm, 0.1 mW) with different spike duration; d) IPSC induced by the light spike of fixed light intensity (635 nm, 1 mW) with different pulse duration; e) change of EPSC and IPSC with different pulse duration. f) EPSC induced by two consecutive light spikes (635 nm, 0.1 mW, 1 s) with a spike interval of 1 s; g) IPSC induced by two consecutive light spikes (635 nm, 1 mW, 1 s) with a spike interval of 1 s; h) PPF of ratio versus interstimulus time for EPSC and IPSC; V_ds_ = 0.01 mV and V_gs_ = 0 V.

The transmission of information in the human brain depends on synapses. **Figure** **4**a is a schematic diagram of signal transmission in an organism through synapses between neurons. After receiving the external stimulus, neurotransmitters will be released from the anterior neuron to the posterior neuron in the form of exocytosis, causing changes in electrical signals. In biological postsynaptic neurons, the synaptic potentials lead to excitatory postsynaptic current (EPSC) or inhibitory postsynaptic current (IPSC), which respectively determine the gain or loss of synaptic weight. The dynamic photoresponse of our device was tested to assess its synaptic behavior. As mentioned in Section 2.1, the device response at V_ds_ = 0.01 mV was similar to that at higher V_ds_. To avoid the influence on I_ds_ from gate bias, we set V_gs_ = 0 V and operate the device using light as the only input energy. From Figure 4b and Figure S7 (Supporting Information), we found that when the laser pulse duration is 5 s, the device took ≈1–2 s to stabilize. Unlike the recently reported photonic synapses, we can switch between EPSC and IPSC for the Gr/WSe_2_ device by changing solely the incident laser power (P_in_). When stimulating the Gr/WSe_2_ device at 635 nm with a P_in_ less than 0.5 mW, we observed EPSC; when the P_in_ is >0.5 and <3 mW, we instead obtained IPSC. Also, we carried out a dynamic photoresponse test for the device at P_in_ = 0.1 and 1 mW with 0.66 s laser pulse duration and observed a typical EPSC and IPSC performance.

Synaptic plasticity is the basic function of the human brain to carry out advanced learning instructions, which can be divided into short‐term plasticity and long‐term plasticity. Short‐term plasticity has a retention time of several hundred milliseconds to a few minutes and plays an important role in the processing or learning of temporary information. As shown in Figure 4c,d, we applied fixed incident light power of 0.1 and 1 mW, respectively with different pulse durations.

Paired pulse facilitation (PPF) is a typical form of short‐term plasticity. After 2s of illumination at 635 nm wavelength with a P_in_ of 0.1 mW, a single EPSC behavior appeared. After 1 s interval, the light pulse under the same conditions was applied again. The secondary current increase value (A_2_) was higher than the primary current increase value A_1_, indicating that the Gr/WSe_2_ vdWH device still retained the primary light stimulation behavior when the second light stimulation was performed (Figure 4e). The biomimetic synaptic behavior of the device can be further characterized by the PPF index, defined as:

(4) $$\mathrm{PPF}=\frac{A_{2}}{A_{1}}\times 100\%$$

where A_1_ is the EPSC amplitude of the first light pulse stimulation, and A_2_ is the EPSC amplitude of the second light pulse stimulation under the same conditions. The PPF values were calculated according to Equation (1) by changing the interval time (Δ*t*) between two successive light pulses from 80 to 160 ms, 490 ms, 660 ms, 1570 ms, and 2480 ms. As can be seen from Figure 4f,g, PPF decreases gradually with the increase of Δt. It is found that the downward trend of PPF can be fitted with a double exponential function (Equation (5))

(5) $$\mathrm{PPF}=C_{1}\exp\left(-\frac{\Delta t}{\tau_{1}}\right)+C_{2}\exp\left(-\frac{\Delta t}{\tau_{2}}\right)+1$$

where *C*_1_ and *C*_2_ represent the fast and slow speed promotion amplitude, while τ_1_ and τ_2_ represent the fast and slow speed characteristic relaxation time, respectively. The red line in Figure 4h is the double exponential function curve fitted according to Equation (2). For EPSC we found that τ_1_ = 0.11 s and τ_2_ = 0.56 s, while for IPSC we obtained 0.49 and 0.49 s respectively, similar to the time scale of biological synapses.^[^ ^23^ ^]^

The potential applications of artificial synapses in neuromorphic circuits limit their energy consumption. Although several remarkable devices and materials simulating synaptic function (electrical or optical) have recently been reported (listed in **Table** 1), the energy consumption is still several orders of magnitude higher than that of biological synapses (≈10 fJ per activity). Our Gr/WSe_2_ optoelectric synapse's average energy consumption (*E*_ave_) during training can be calculated from the training voltage (V), the delta value of conductance before and after training (Δ*G*), the duration of the light spike (t), and the number of spikes (N). Thus, *E*_ave_ = (V^2^ × ΔG × t)/N = (V × Δ I_dark_ × t)/N, where ΔI_dark_ = 0.159 nA, *t* = 80 ms, V = 0.01 mV, N = 1. It results in >0.4% conductivity change (high enough to simulate all synaptic functions) with an average active area of 15.2 µm^2^ with an illuminating power of 0.1 mW and duration of 80 ms µm^−2^, consuming *E*_ave_ ≈ 127 aJ for each synaptic activity. The results indicate that the photogating effect contributes to the synaptic behavior, and the photovoltaic effect is used to convert the energy of the photons to electrical energy and make the power consumption of the device ultralow.

**Table 1:** Comparison of energy consumption of various optoelectrical synaptic devices.

Devices	λ [nm]	EPSC/IPSC [nA]	1st PPF ratio	Modulation of potentiation and depression	Power consumption	Ref.
Gr/WSe_2_	635	6	1.5 @Δ*t* = 80 ms	single wavelength light‐potentiation and depression	127 aJ	This work
TIPS‐pentacene/PS OTFT	450	3.39	1.7 @Δ*t* = 50 ms	light‐potentiation /electric‐depression	36 aJ	[24]
Black phosphorus /CdS	450	1250	/	light‐potentiation /electric‐depression	4780 aJ	[25]
POx/black phosphorus	280,365	≈100	≈0.3 @Δ*t* = 100 ms	285 nm light‐potentiation/365 nm light‐depression	3.5e6 aJ	[26]
Ag‐TiO_2_	350–650	≈300	≈1.8 @Δ*t* = 10 µs	Visible light‐potentiation/UV light‐depression	≈2.4e10 aJ	[27]

## 3 Conclusion and Perspective

In summary, we demonstrated a photogating mechanism for Gr/WSe_2_ vdWH optoelectronic synaptic devices that offers switching between NPC and PPC with ultralow electrical power consumption, relying solely on the intensity of the optical stimuli. Through careful characterization, we elucidated the complex interplay of photovoltage and photogating at the different interfaces. We first showed that the switching behavior depends on the sign of the effective drain‐source voltage $V_{\mathrm{ds}}^{\mathrm{eff}}\;$ and requires only small V_ds_ to occur. Second, the contribution of photogating across the multiple interfaces of the Gr/WSe_2_/SiO_2_/Si structure was deconvoluted. The results show that gate bias and laser power can modulate the sign and magnitude of photocurrent because the photogating effects in the trap centers induce different levels of band bending in each interface. Notably, when a very small V_ds_ is applied, the response time of the device slows down and presents a synaptic behavior. Consequently, we confirmed and quantified typical synaptic functions, including EPSC, IPSC, and PPF. Moreover, the synaptic plasticity was successfully modulated by varying the parameters of light spikes. It is worth noting that it has an extremely low electrical power consumption of ≈127 aJ because of the photovoltaic effect induced by the Schottky contact between Gr and metal. Overall, this work provides a strategy to achieve all‐optical modulation of neuromorphic devices with a single monochromatic light source. It could therefore advance the development of integrated neuromorphic computing systems.

## 4 Experimental Section

### Device Fabrication

WSe_2_ flakes (2D Semiconductors Int.) were mechanically exfoliated and then transferred to 300 nm SiO_2_/Si substrates, where Si can be used to apply a gate bias (V_gs_). Subsequently, the WSe_2_ multi‐layer flake was etched into a 15 um×5um rectangle through photolithograph and XF_4_ gas etching. Monolayer graphene was ordered from Graphenea Int., and a typical polymethyl methacrylate‐assisted wet transfer method was used to stack graphene over the WSe_2_. After wet transferring, the monolayer graphene was etched into a ribbon with length and width of 20 and 10 µm through photolithograph and O_2_ plasma dry‐etching. Finally, laser writing (MLA150, Heidelberg Instruments Int.) and e‐beam evaporation (LAB 600H, Leybold Optics) were used to define contacts to the heterostructures, where source (s) and drain (d) Ti/Au (5/50 nm) contacts are evaporated to contact the graphene ribbon. (see Figure S8; Section S5, Supporting Information).

### Device Characterization

The Raman and photoluminescence (PL) spectra were obtained via Raman Microscope (Renishaw Ltd. Co., inVia) using an excitation wavelength of 532 nm. The flake thickness and topography were tested by AFM (Bruker, FastScan). The photoelectrical characterization is done by using a home‐built set‐up (see Figure S9 and Section S6, Supporting Information), which consists of a black sealed box, several optical paths, a motorized probe station (miBot, Imina Technologies), a camera, and two Keithely 2450 source meters. The source meters were used for applying source‐drain bias (V_ds_) and source‐gate bias (V_gs_). To reduce the thermoelectric effect induced by electrical tests, the delay of sweeping V_ds_ and V_gs_ was set as 1 ms. Because the samples are very sensitive to ambient moisture, we stored them in the N_2_ gas tank before and after photoelectrical tests. During tests, dry N_2_ with a low flow rate was injected into the black sealed box to keep it dry.

## Conflict of Interest

The authors declare no conflict of interest.

## Supporting information

Supporting Information

## Data Availability

The data that support the findings of this study are available from the corresponding author upon reasonable request.
