# Supplementary material for: Light‐Intensity Switching of Graphene/WSe2 Synaptic Devices
Source: Adv Sci (Weinh). 2024 Apr 22;11(24):2309876. doi: 10.1002/advs.202309876 (PMC11199970; doi:10.1002/advs.202309876)
Supplement: Supplementary file 1 — Supporting Information [file ADVS-11-2309876-s001.pdf]

ADVANCED SCIENCE
Open Access

Supporting Information

for *Adv. Sci.*, DOI 10.1002/advs.202309876

Light-Intensity Switching of Graphene/WSe_2 Synaptic Devices

*Hongyu Tang, Tarique Anwar, Min Seok Jang and Giulia Tagliabue**

Supplementary information for

Light-Intensity Switching of Graphene/WSe_2 Synaptic Devices

Hongyu Tang, [1,†] Tarique Anwar, [1] Min Seok Jang,[2] Giulia Tagliabue [1,*]

[1]Laboratory of Nanoscience for Energy Technologies (LNET), École Polytechnique Fédérale de Lausanne, Station 9, CH-1015, Lausanne, Switzerland.

[2]School of Electrical Engineering, Korea Advanced Institute of Science and Technology, Daejeon, Korea,

† Current affiliation: Academy of Engineering & Technology, Fudan University, Handan Road 220, 200433, Shanghai, China

* Corresponding author. Email: giulia.tagliabue@epfl.ch (Giulia Tagliabue)

Table of Contents

S1 – I_{ds}-V_{ds} response at zero gate bias and under different illumination power.

S2 – Photovoltage under different illumination power and V_{bi} after illumination.

S3: I_{ds}-V_{gs} shift of the device after illumination.

S4 – Modelling the effect of impurity density on the carrier transport in graphene.

S5 – Fabrication process.

S6 – Photoelectronic test system.

S1: I_{ds}-V_{ds} response at zero gate bias and under different illumination power.

The Dark0 is the initial dark current, and Dark(i), i=1,2,3,…,7 indicates the other dark current after illumination of respective laser power.

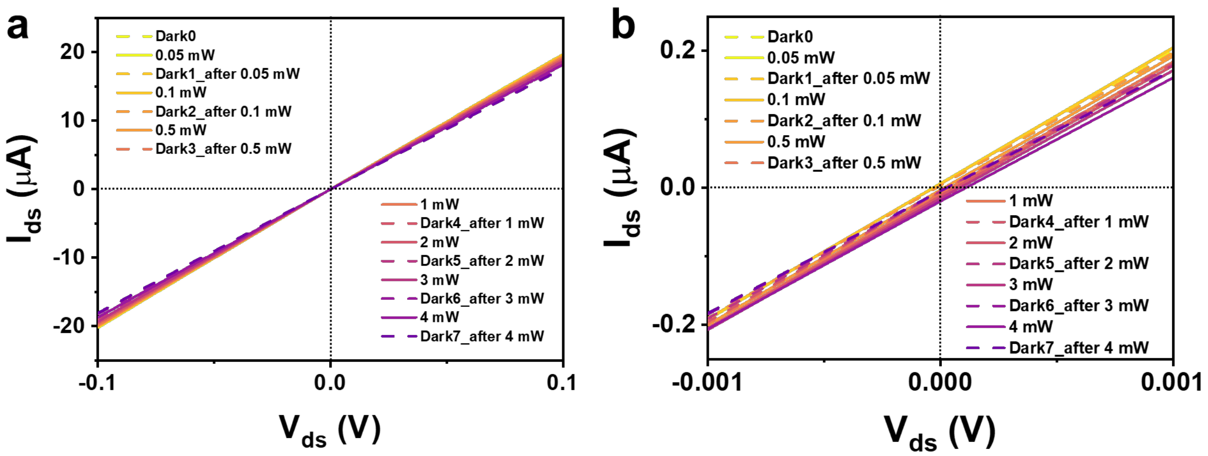

Figure S1: a) I_{ds}-V_{ds} and b) the enlarged curve after and during illumination of different power.

S2: Photovoltage under different illumination power and V_{bi} after illumination

In the photovoltaic effect, photogenerated e–h pairs are separated by an internal electric field that originates from the Schottky barrier at the interface between graphene and metal. The photovoltage (V_{PV}) of the Gr/WSe_2 vdWH can be calculated from the output curve under illumination, as shown in **Figure S2**. The intercept of the I_{ds}-V_{ds} curve indicates the open-circuit voltage, that is the photovoltage. The slope of the I_{ds}-V_{ds} curve indicates the built-in electrical potential difference (V_{bi}). It is found that the V_{bi} is ~0.2 mV and the V_{PV} is in the range of 0.4-0.6 mV. Due to asymmetry of the drain and source, there is a non-zero photovoltage $V_{PV} \approx \Delta V_D - \Delta V_S$, and a resultant photocurrent even at $V_{ds} = 0$[1]. The band diagram at the graphene-metal interface is shown in **Figure S2**(c). Furthermore, to provide a theoretical outlook, the potential step ΔV is given by

$$\Delta V = sgn\left(V_{gs} - V_{dirac}\right)\hbar v_f \sqrt{\pi\alpha\left|V_{gs} - V_{dirac}\right|} - \Delta E_{fm}$$

$$I_{ph} \propto (n_D^* \Delta V_D - n_S^* \Delta V_S)$$

where $\alpha = 7.2 \times 10^{10} cm^{-2} V^{-1}, \hbar v_f = 5.52 eV\text{Å}$, ΔE_{fm} is the fermi level shift of metal contacted graphene and n^* is the photogenerated carrier.

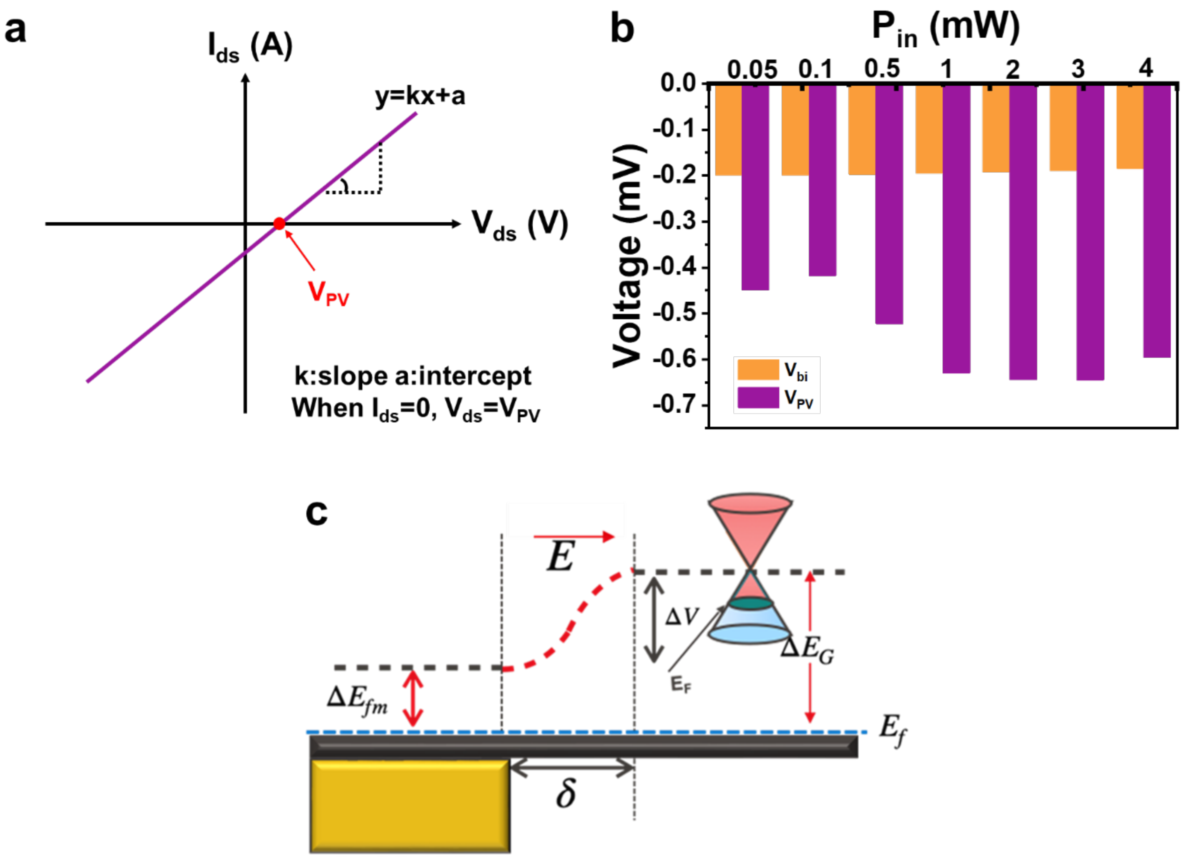

Figure S2: Photovoltage (V_{PV}) and built-in electrical potential difference (V_{bi}) induced by drain-source asymmetry under different optical power determined from the intercept and slope of the I_{ds}-V_{ds} curve. c) Band-bending resulting in photovoltage along the lateral junction.

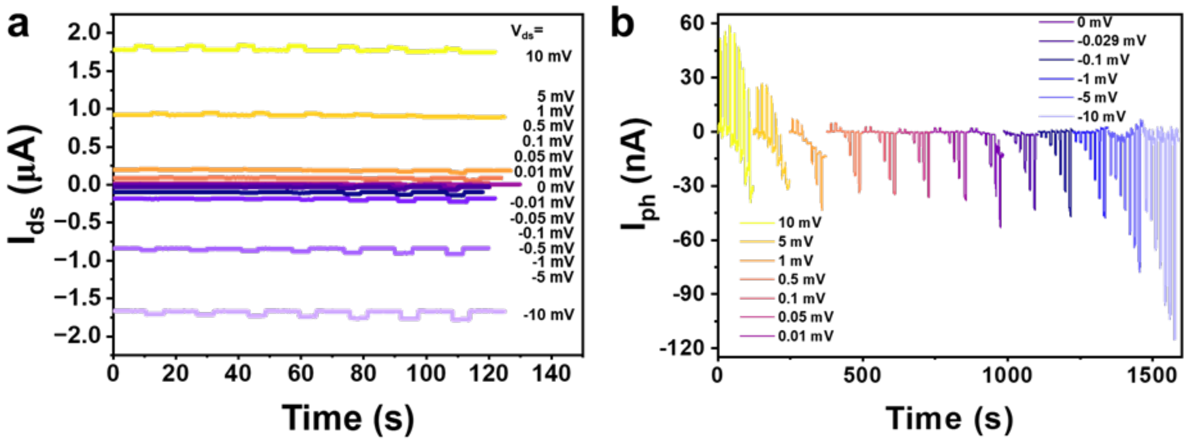

Figure S3: Dynamic a) I_{ds} and b) I_{ph} change at V_{gs}=0V and different P_{in} for a series of V_{ds}.

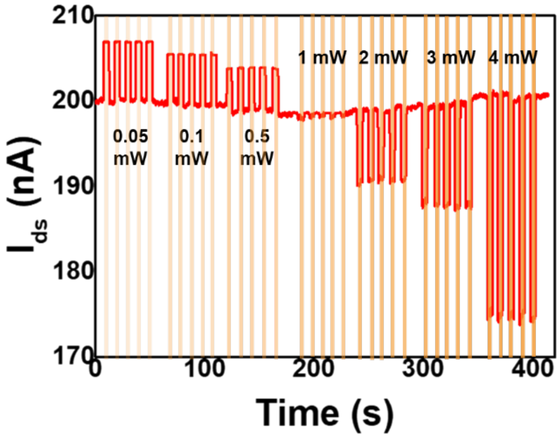

Figure S4: The repeatability of the dynamic photocurrent current change at V_{gs}=0V and different P_{in} for a constant Vds=1 mV.

S3: I_{ds}-V_{gs} shift of the device after illumination.

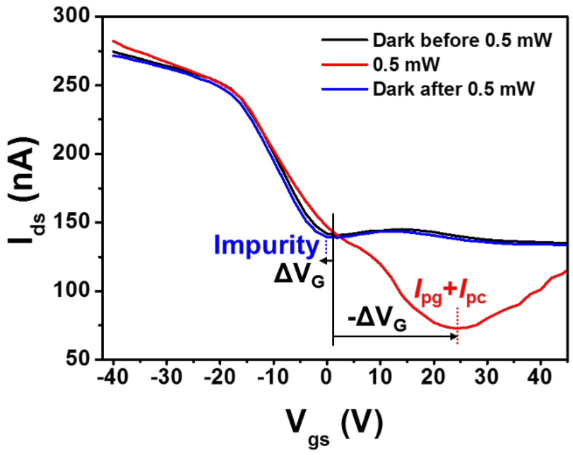

Figure S5. I_{ds}-V_{gs} shift after illumination. Dark line, red line, and blue line represent the dark current before illumination, the current under illumination at P_{in}= 0.5 mW, and the dark current after illumination, respectively.

S4: Modelling the effect of impurity density on the carrier transport in graphene.

The carrier transport in graphene in presence of scattering from charged impurities was modelled based on the work of Adman et al. [2]

$$\frac{n^*}{n_{imp}} = 2r_s^2 C_0\left(r_s = 0.8, a = 4d\sqrt{\pi n^*}\,\right)$$

$$\bar{n} = \frac{n_{imp}^2}{4n^*}$$

where $\quad C_0 = -1 + \frac{4E_1(a)}{(2+\pi r_s)^2} + \frac{2e^{-a}r_s}{1+2r_s} + (1+2r_s a)e^{2r_s a}(E_1[2r_s a] - E_1[a(1+2r_s a)]$

and the exponential integral $\quad E_1(z) = \int_z^\infty t^{-1}e^{-t}dt$

The Dirac point shift depends on impurity density, which is given as:

$$\boldsymbol{V_{dirac} = e\bar{n}C_{ox}}$$

where, C_{ox} is the capacitance per unit area of the dielectric layer (SiO_2). C_{ox}=ε/d, d being the thickness of the dielectric, which is equal to 300nm. Thus, the carrier density n is given by:

$$n = \sqrt{n_g^2 + 4n_{min}^2}$$

$$n_{min} = \sqrt{\left(n^*/2\right)^2 + 4n_{th}^2}$$

where n_{th} is the thermal/intrinsic carrier concentration and n_g is the carrier density change due to applied gate bias, V_g.

$$n_{th} = \frac{\pi}{6}\left(\frac{k_B T}{\hbar v_f}\right)^2$$

$$n_g = \frac{c_{ox}\left(V_g - V_{dirac}\right)}{e}$$

The change in mobility due to change in impurity, density can be expressed as[3]:

$$\boldsymbol{\frac{\mu}{\mu_{ref}} = \frac{n_{imp,ref}}{n_{imp}}}$$

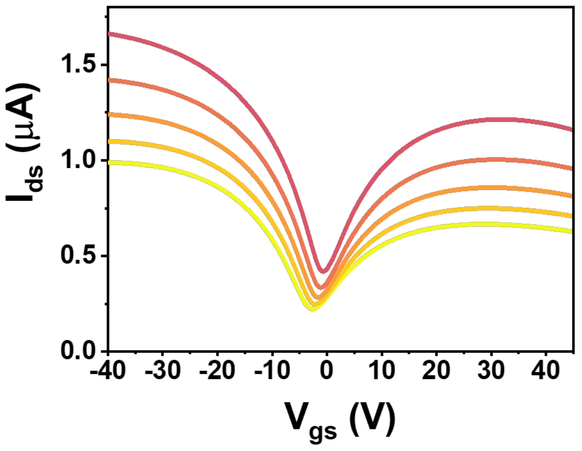

Figure S6. Simulated transfer curve for the dark current I_{ds} by increasing the impurity density for carrier scattering that affects the conductance of graphene channel.

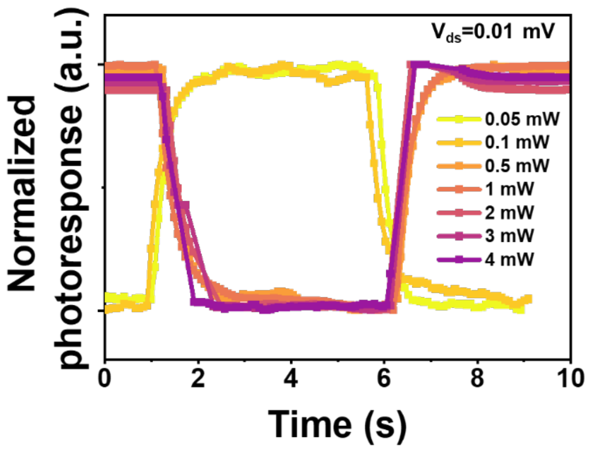

Figure S7. Normalized photoresponse of the synaptic device at different P_{in} for a constant V_{ds}=0.01 mV.

S5: Fabrication process

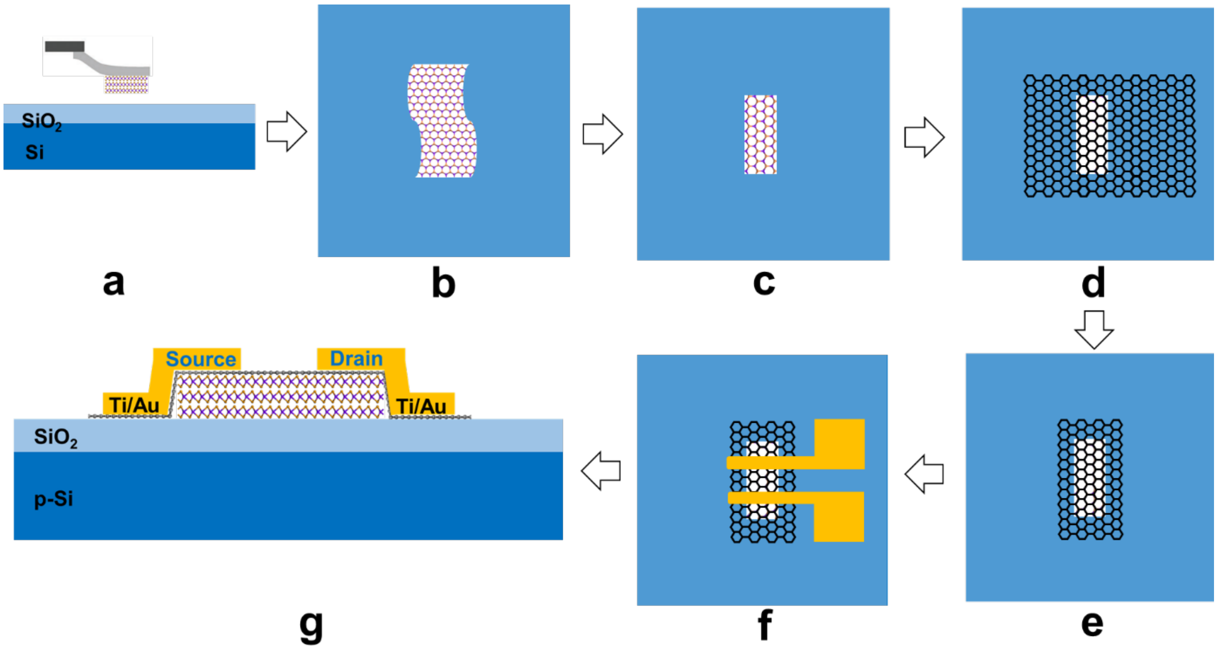

Figure S8. Fabrication process flow of Gr/WSe_2 vdWH devices. a) Dry-transferring mechanical exfoliated WSe_2 multi-layer flake to the SiO_2/Si substrate. b) WSe_2 with random shape is on the surface of SiO_2/Si substrate. c) Patterning the WSe_2 flake into a 15 um×5um rectangle through photolithograph and XF_4 gas etching. d) Wet-transferring monolayer graphene over the WSe_2. e) Patterning the monolayer graphene to a ribbon with length and width of 20 μm and 10 μm through photolithograph and O_2 plasma dry-etching. f) Laser writing and e-beam evaporation are used to define contacts to the heterostructures, where source (s) and drain (d) Ti/Au (5 nm/50 nm) contacts are evaporated to contact the graphene ribbon. (g) Cross section of Gr/WSe_2 vdWH devices.

S6: Photoelectronic test system

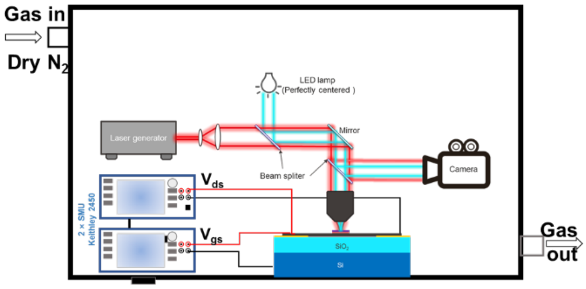

Figure S9. Photoelectronic test system

References

[1] E. J. H. Lee, K. Balasubramanian, R. T. Weitz, M. Burghard, K. Kern, *Nat Nanotechnol* **2008**, *3* (8), 486, https://doi.org/10.1038/nnano.2008.172.

[2] S. Adam, E. H. Hwang, V. M. Galitski, S. Das Sarma, *P Natl Acad Sci USA* **2007**, *104* (47), 18392, https://doi.org/10.1073/pnas.0704772104.

[3] J. H. Chen, C. Jang, S. Adam, M. S. Fuhrer, E. D. Williams, M. Ishigami, *Nat Phys* **2008**, *4* (5), 377, https://doi.org/10.1038/nphys935.
